# The investigation of the efficacy and safety of stromal vascular fraction in the treatment of nanofat-treated acne scar: a randomized blinded controlled clinical trial

**DOI:** 10.1186/s13287-022-02957-2

**Published:** 2022-07-15

**Authors:** Elham Behrangi, Sepideh Moradi, Mohammadreza Ghassemi, Azadeh Goodarzi, Amirreza Hanifnia, Sona Zare, Maryam Nouri, Abbas Dehghani, Azadeh Seifadini, Mohammad Ali Nilforoushzadeh, Masoumeh Roohaninasab

**Affiliations:** 1 Department of Dermatology, Rasool Akram Medical Complex Clinical Research Development Center (RCRDC), School of Medicine, Iran University of Medical Sciences, Tehran, Iran; 2 Skin and Stem Cell Research Center, Tehran University of Medical Sciences, Tehran, Iran; 3 Skin Repair Research Center, Jordan Dermatology and Hair Transplantation Center, Tehran, Iran; 4 Laser Application in Medical Sciences Research Center, Shahid Beheshti University of Medical Sciences, Tehran, Iran

**Keywords:** Acne vulgaris, Scar, Lipotransfer, Fat injection, Nanofat injection, Stromal vascular fraction, Trial

## Abstract

**Background:**

Acne is the most common skin disorder which is known as a chronic inflammatory disease with psychological burden and reduced quality of life. Adipose tissue-derived stromal vascular fraction (SVF) is recognized as a source of regenerative cells and improves the quality of skin by increasing collagen content. To date, a few studies have been performed on the therapeutic role of SVF in the treatment of acne scars.

**Methods:**

This randomized, single-blinded clinical trial was performed on 7 patients with acne scars. In all patients, the initial grade of acne (volume, area and depth) was evaluated and ultrasound of the relevant scar was performed to evaluate neocollagenesis. As a spilt face study, for treating the scars, we used nanofat subcutaneously on one side of the face (control group) and combination of nanofat subcutaneously and SVF intradermally on the opposite side (intervention group). The patients were evaluated for severity of acne by visioface after one month, also for thickness of epidermis and dermis by ultrasound after one month and three months.

**Results:**

All of the apparent findings of scars improved in two groups after one month, but these changes were significant just for the group treated with SVF (*p* value < 0.05). Epidermal, dermal and complete thicknesses during the first month in both control and intervention groups were significantly increased (*p* value < 0.05) but between the first and third months, there was no significant difference in the variables (*p* value > 0.05). The findings showed that dermal and complete thicknesses of the skin in the first month were different between two groups significantly (*p* value: 0.042 and 0.040, respectively).

**Conclusion:**

The use of SVF in the treatment of patients with acne scars accelerates the improvement of volume, area and depth of the scar by increasing collagen content and the dermal thickness, so it can be used as a potentially effective treatment for these patients.

## Introduction

Acne is one of the most common skin disorders in the world which involves millions of people in all ages and races each year [1]. This complication is a chronic inflammatory disease which is characterized by the presence of polymorphic skin lesions such as papules, pustules, nodules, or cysts. Acne in addition to beauty problems is associated with psychological consequences and affects the quality of life [2, 3]. Acne scar is the result of a severe inflammatory process that is associated with dermal involvement, but sometimes it is the outcome of manipulation of severe acne lesions by the patients themselves [4, 5]. The different treatments for scars have been considered. The topical retinoids are effective in the treatment of inflammatory and non-inflammatory lesions by preventing comedones, reducing existing comedones and targeting inflammation. Topical and oral antibiotics are effective as monotherapy, but they are more effective in combination with topical retinoids. Adding benzoyl peroxide to antibiotic therapy reduces the risk of bacterial resistance. Oral isotretinoin has been approved for the treatment of severe acne [6]. Despite numerous topical and systemic treatments for acne, there are still many problems in the treatment of these lesions, especially in the repair of acne scars [7].

In general, acne scar therapeutical strategies are based on physical approaches (including laser, pulse light and cryotherapy), surgical approaches (dermabrasion, punk oxygen), or types of topical drugs medications [2]. However, almost the majority of these therapeutical methods have remained inconclusive and unsatisfactory and in fact no gold standard treatment has been provided for these lesions. Meanwhile, autologous fat grafting has been used as a comprehensive method for tissue repair in the recent decade, and in some studies [8–10], the effectiveness of this method in repairing the structure and formation of the skin and rejuvenation has been confirmed [11–14]. Fat tissue consists of two main components which are as follows: mature adipocyte cells and stromal vascular fraction (SVF) [15–17]. SVF is composed of heterogeneous cell populations including blood-derived cells (CD45 +), adipose-derived adherent stromal cells (ASCs: CD31-, CD34 + , CD45-, CD90 + , CD105-, CD146-), endothelial (progenitor) cells (CD31 + , CD34 + , CD45-, CD90 + , CD105low, CD146 +), pericytes (CD31-, CD34-, CD45-, CD90 + , CD105-, CD146 +) and other cells.

The most important feature of these SVF cells is their high repairability due to the presence of stem cells in them, interacting and interfering with growth factors [18–20]. Various studies show that this method has been effective in improving scars in a large number of patients [21, 22]. Previous findings demonstrated that the application of SVF can improve tissue healing and maintenance of fat graft volume. In fact, by early methods of adipose tissue transplantation, significant portions of the transplanted tissue were often reabsorbed, perhaps for a poor blood supply due to insufficient neoangiogenesis. Therefore, to decrease the graft failure rate, different strategies have been developed in the last years to obtain fat grafts as rich as possible of mesenchymal stem cells, exploiting their regenerative capacities [15, 19, 23–25].

The use of this method in the treatment of skin wrinkles and hyperpigmentation is well understood; however, scar treatment is one of the leading challenges for dermatologist professionals and a few studies have been conducted regarding the therapeutic effects of this technique in the treatment of acne scars [13, 21, 22, 25]. The present study was performed with the aim to investigate the efficacy and safety of using stromal vascular fraction in the nanofat-treated acne scars.

## Materials and methods

### Patients

This randomized, single-blinded clinical trial study was performed on 7 patients with acne scars referred to a dermatology clinic from August to December 2021. At the beginning of the study, patients' background information was prepared and recorded in the study checklist. In all patients, the initial grade of acne was determined qualitatively based on the Goodman and Baron grading system and the severity of acne (volume, area and depth) was determined by imaging. Also, before the intervention, ultrasound of the relevant scar was performed to examine neocollagenesis.

Prior to the intervention, a written consent was obtained from the patients and to be assured them that the therapeutic interventions were not associated with complications and no cost was imposed to them. Then, the patients were divided into a group of 7 people based on a random table of numbers. In each patient, one part of the face was selected as the intervention area and the other part as the control.

The inclusion criteria for the study were individuals aged 18 to 70 years old with clinical diagnosis of acne scar. The exclusion criteria of the study were included family history of bleeding or coagulant diseases, consumption of anticoagulants or steroids drugs, blood hemoglobin less than 10 and platelets less than 150, the presence of active skin or systemic infection, malignancy or history of receiving chemotherapy, breastfeeding or pregnancy and history of receiving any analgesic medication during one week prior to the intervention.

### Randomization and blinding

The patients were divided into two groups according to the generated randomized list using the relevant software in a randomized block method with a volume of 7 people. The side of the face is selected based on a random selection of a box with twenty sealed envelopes for each patient which includes code A or B (right side of the face with SVF injection: A and right side of the face without injection: B).

### SVF cells isolation

At the first, 100 cc of fat was received from the thigh area. To remove most red blood cells and leukocytes, the tissue was washed with phosphate-buffered saline (PBS) (Miltenyi Biotec, Cologne, Germany) and fat tissue digested with type I collagenase (Worthington Biochemical Corp., Lakewood, USA) at 37° C, 20 min, so solution of collagenase, final concentration of 0.1%, was made. Enzyme digestion was prevented by washing with DMEM 10% FBS (Invitrogen, Carlsbad, USA), floating and lysed adipocytes were waste, and cells of the SVF were pelleted by 10 min centrifugation at 500 g. The pellet was suspended in the PBS, and an erythrocyte lysis buffer (Sigma-Aldrich Corp, St. Louis, USA) was added and incubated 10 min at 37 °C. This cell suspension was centrifuged (500 g, 5 min), and cells were counted.

### Intervention methods

In these patients, after preparing SVF and nanofat taken from the fat, the scars were treated on one side of the face with nanofat subcutaneously (control group) and on the other side of the face with a combination of nanofat subcutaneously and SVF intradermally (case group).

### Assessment methods

Post-treatment evaluation of patients was performed on the basis of following description:

1. Scar size: We evaluated the severity of scars in two groups by determining scar size variables including volume, area and depth based on digital re-imaging after one month from the intervention.
2. Dermal thickness: Since SVF injection can stimulate fibroblasts for producing collagen, we used ultrasound in order to determine collagen content and neocollagenesis affecting on epidermal and dermal thickness and complete thickness of the skin after one month and three months from the intervention.
3. Acne scar grade: Determination of qualitative grade of acne scar based on Goodman and Baron system (grade 1 to grade 4).
4. Physician satisfaction: Determination of the level of treating physician satisfaction from improving the scars including: low response (less than 30%), moderate response (30% to 50%), good response (50% to 70%) and excellent response (above 70%),
5. Patient satisfaction: Determination of the level of patient satisfaction from treatment results including: low response (less than 30%), moderate response (30% to 50%), good response (50% to 70%) and excellent response (above 70%).

Finally, these complications were compared between scars on both sides of the face. The data were gathered with the help of pre-prepared checklists.

### Data analysis

The quantitative variables were expressed as mean and standard deviation (mean ± SD), and stratified qualitative variables were expressed as percent. The *t* test or ANOVA was used to compare quantitative variables, and Chi-square test was used to compare qualitative variables. The significance level was considered less than 0.05. In order to data statistical analysis, SPSS software version 25 was used.

### Ethical principles

All of the collected information was kept confidential and analyzed without a specific name. The present participants in this project were adhered to all Helsinki ethical principles (IRCT20200901048586N1). This research was approved by the Research Council with the ethics code number IR.IUMS.FMD.REC.2021.291.

## Results

Among 7 studied patients, four SVF injections were performed on the right side of the face and 3 injections were performed on the left side. The side where the SVF was not injected was considered as a control in analyses. The basic characteristics of the variables were statistically equal to each other and prior to the intervention, and the variables related to scar on the injection side (experimental group) and on the opposite side (control group) had not significant difference from each other (*p* value > 0.05) (Table 1).

**Table 1:** The basic variables of scars in the injected (case) and non-injected (control) sides of patients' face

Groups	Mean	*p* value
*Volume*		
Control	134.44	0.94
Case	131.90	
*Area*		
Control	11.42	0.68
Case	12.65	
*Depth*		
Control	10.85	0.19
Case	9.85	
*Epidermal thickness*		
Control	67.71	0.80
Case	66.42	
*Dermal thickness*		
Control	687.71	0.34
Case	629.42	
*Complete thickness*		
Control	755.42	0.34
Case	695.85	

*Independent *t* test

The apparent findings related to visual face in the case group including volume, area and depth had decreased significantly (*p* value < 0.05) in time comparison, as it is observed in Table 2. In the first month, epidermal thickness, dermal thickness and complete thickness in the case group (face injected side) were significantly improved (*p* value < 0.05), so during the same month, all measured variables, including apparent and sonographic variables in the face injected side group, had a significant improvement.

**Table 2:** Comparison of acne variables in case and control groups during one and three months

	Baseline		After 1 month		After 3 months		Change% *p* value		
	Mean	SD	Mean	SD	Mean	SD	Baseline to Month1	Baseline to Month3	Month1 to Month3
*Volume*									
Control	134.45	78.88	99.86	40.97	–	–	− 18.12% 0.112	–	–
Case	131.90	63.04	84.23	44.41	–	–	− 33.87% 0.026	–	–
*Area*									
Control	11.43	5.52	9.36	3.69	–	–	− 14.97% 0.069	–	–
Case	12.66	5.68	8.43	3.87	–	–	− 30.03% 0.033	–	–
*Depth*									
Control	10.86	1.57	10.14	0.90	–	–	− 5.06% 0.310	–	–
Case	9.86	1.07	9.14	0.90	–	–	− 6.88% 0.047	–	–
*Epidermal thickness*									
Control	67.71	8.69	78.28	7.91	83.75	21.03	+ 16.17% 0.001	+ 33.75% 0.158	+ 12.91% 0.360
Case	66.43	9.91	85.43	12.82	85.25	8.46	+ 30.24% 0.009	+ 41.95% 0.064	+ 9.50% 0.378
*Dermal thickness*									
Control	687.71	110.53	801.14	111.90	893.75	47.89	+ 17.89% 0.033	+ 29.59% 0.042	+ 10.40% 0.119
Case	629.43	111.49	965.57	155.36	981.25	78.57	+ 58.81% 0.008	+ 61.27% 0.002	+ 11.11% 0.101
*Complete thickness*									
Control	755.43	112.47	879.43	113.30	977.50	66.14	+ 17.65% 0.028	+ 29.75% 0.045	+ 29.75% 0.093
Case	695.86	114.65	1051.00	161.84	1066.50	71.39	+ 55.45% 0.007	+ 58.99% 0.001	+ 10.89% 0.101

*p* numbers based on paired *t* test

In the first month, in the control group, sonographic findings had a significant difference from the baseline (*p* value < 0.05), but in the apparent findings, despite improving, they had not significant difference with the baseline (*p* value > 0.05). Compared to the results of the first month, after passing three months from the intervention, there was no significant difference in the sonographic variables measured on both sides of the patients' face (*p* value > 0.05).

At the end of the third month, only sonographic criteria were examined which in case and control groups, both dermal thickness and complete thickness of the skin were significantly better than the baseline, but epidermal thickness had not significant difference in two groups despite improvement.

As it can be seen in Table 3, in the intergroup comparison of variables at different times, dermal thickness and complete thickness of the skin in the first month were improved in the case group compared to the control group and these differences were significant (0.042 and 0.040, respectively), but no significant difference was observed in the third month for the dermal and complete thicknesses of the skin between two groups. Also, the difference in epidermal thickness in the first and third month and apparent variables (volume, area and depth) at the first month was not significant between case and control groups.

**Table 3:** Comparison of scar variables over time between the two sides of the face (case and control groups)

Parameters	Baseline		After 1 month		After 3 months	
	Mean	SD	Mean	SD	Mean	SD
*Volume*						
Control	134.45	78.88	99.86	40.97	–	–
Case	131.90	63.04	84.23	44.41	–	–
*p* value	.948	.507				
*Area*						
Control	11.43	5.52	9.36	3.69	–	–
Case	12.66	5.68	8.43	3.87	–	–
*p* value	.689	.655				
*Depth*						
Control	10.86	1.57	10.14	0.90	–	–
Case	9.86	1.07	9.14	0.90	–	–
*p* value	.190	.060				
*Epidermal thickness*						
Control	67.71	8.69	78.28	7.91	83.75	21.03
Case	66.43	9.91	85.43	12.82	85.25	8.46
*p* value	.801	.233	.899			
*Dermal thickness*						
Control	687.71	110.53	801.14	111.90	893.75	47.89
Case	629.43	111.49	965.57	155.36	981.25	78.57
*p* value	.345	.042	.106			
*Complete thickness*						
Control	755.43	112.47	879.43	113.30	977.50	66.14
Case	695.86	114.65	1051.00	161.84	1066.50	71.39
*p* value	.346	.040	.117			

*Independent *t* test

**Fig. 1:**
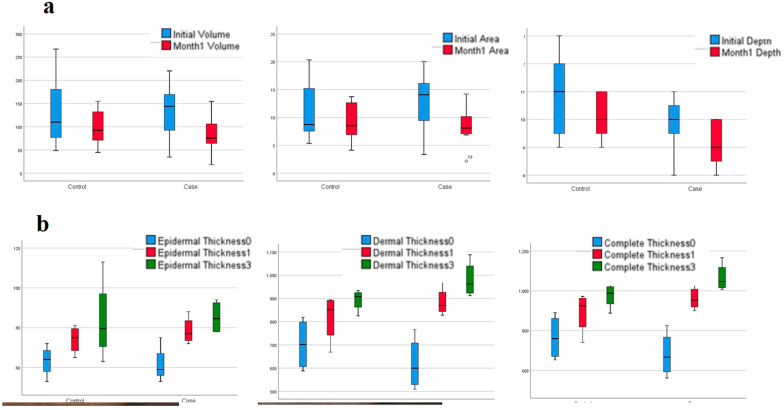
**a** Comparison of the apparent variables of scars over time in the experimental and control positions of patients' face. **b** Comparison of sonographic variables of scars over time in the case and control positions of patients' face

**Fig. 2:**
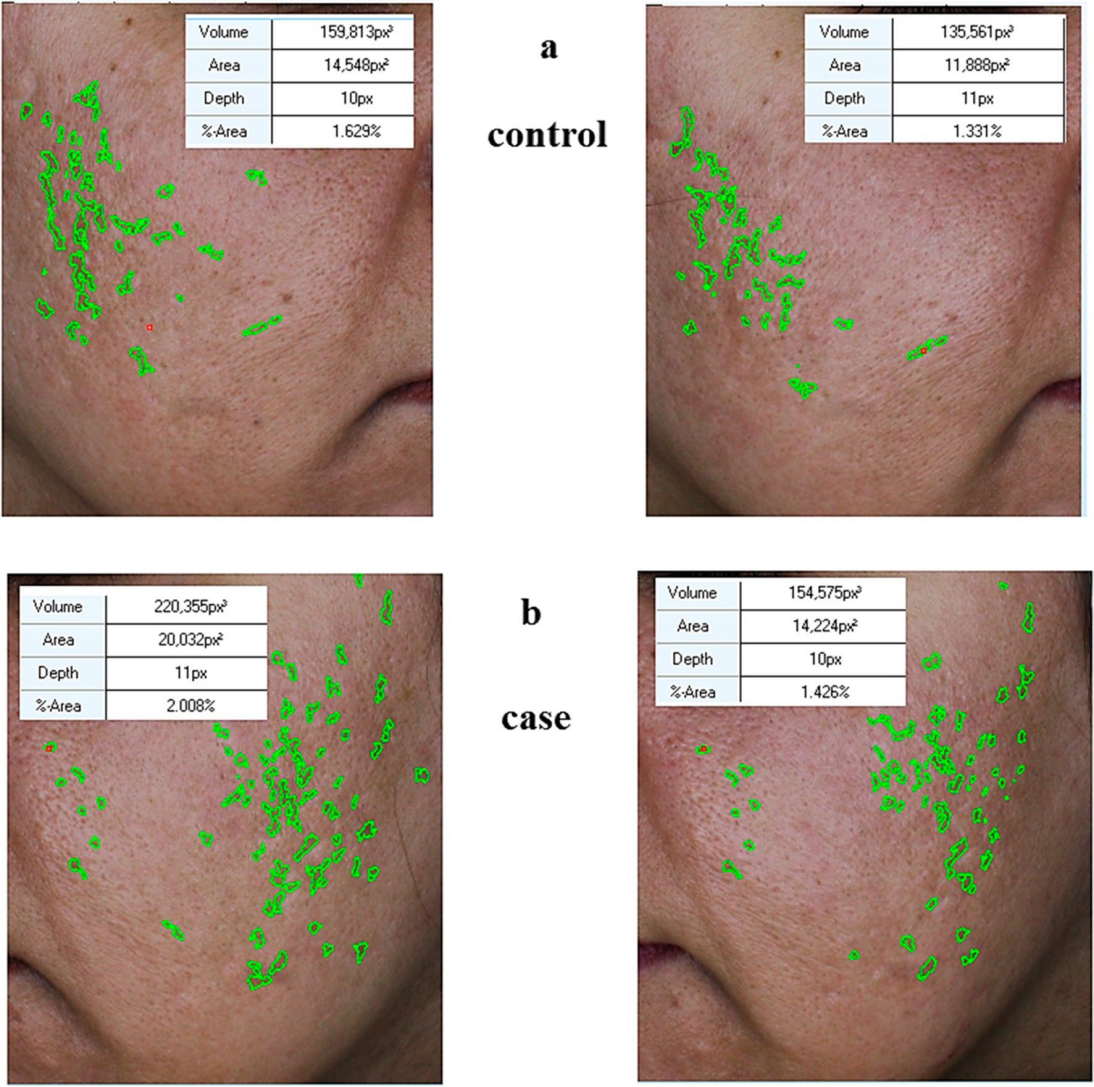
**a** Patients’ recovery period in both experimental and control groups, **b** patients’ recovery course in control group (non-injected side)

A comparison of the apparent and sonographic changes of scars over time can be seen in Fig. 1A, B. Also, changes in facial appearance in the injection and control positions can be seen in Fig. 2A, B.

**Fig. 3:**
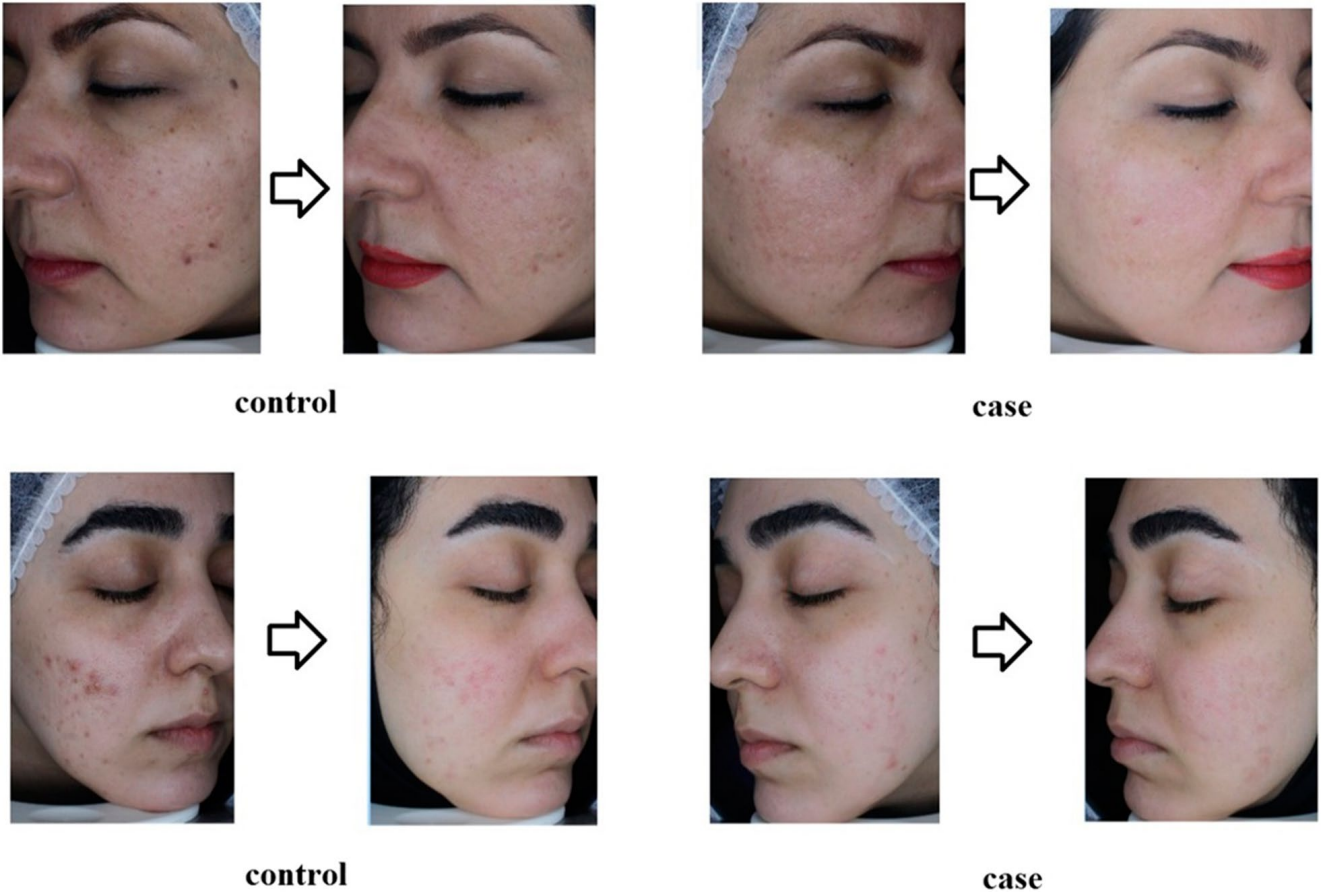
The extent of facial changes in the injection and control positions

The extent of facial changes in the injection and control positions can be seen in Fig. 3. According to the images taken in the case group, the amount of improvement can be clearly seen. It can be concluded that during the time, the improvement has occurred on both sides with and without facial injection, but the rate of this improvement was faster on the injected side (with SFV) than the opposite side.

## Discussion

Regarding the previous studies, fat grafting, adipose-derived mesenchymal stem cells (AD-MSCs) and SVFs have played a role in improving the hair regrowth [26–28], wound healing [9, 10, 22] and tissue regeneration [29, 30] when used alone or in combining with hyaluronic acid, biomaterials, platelet-rich plasma [10, 31, 32], and also, AD-MSCs have been used in inhibiting COVID-19 recently [33, 34].

In previous researches, the therapeutic function of SFV in improving skin fibrosis and scar-related complications has been identified [21, 25, 35]. This study has paid much attention to investigate the effect of SFV on acne scar in terms of case–control. Our findings showed that finally, epidermal thickness, dermal thickness and complete thickness during one month in the control and intervention groups have improved significantly. But in both groups, no significant difference was observed during the one-month period with three-month period. In addition, in an intergroup comparison, there was a significant difference between dermal thickness and complete thickness in the first month between the case and control groups. The findings of this research were consistent with similar studies [25, 35].

In 2018, Shan et al. evaluated the effect of injecting adipose stem cells (ASCs) from SVF cell solution into the treatment of acne vulgaris scars. Their findings showed that the infiltration of inflammatory cells in weeks 2 and 4 was significantly decreased after injection and also re-epithelialization improved in the SVF injection group. In addition, the levels of inflammatory factors such as TNF-α and IL-1α and the protein levels of MMP-2 and K16 were decreased. They stated that there is a significant treatment for acne vulgaris scars, since that conditioned media plays a key role in the treatment. This subject can be used as a therapeutic method to regenerate and treat the scars [35]. However, in the present study, sonographic variables such as dermis, epidermis and total density lost their difference with baseline state after 3 months. This lack of significance, of course, can be related to the low number of samples that ultrasound was performed three months later and the sample size was limited in this regard.

In another co-study by Gentile et al., three different nanofat procedures, including supercharged, evo and centrifuged, were compared with the classical nanofat method. In this study, it was found that the supercharged-modified nanofat method generates the best results in terms of clinical results and SVF performance. This study showed the effectiveness of SVF well in wound healing [25]. In the present study, the apparent variables of the wound such as its volume, area and depth on the SVF-treated side improved significantly during one month, but such an improvement did not occur on the face control side. This significant difference over time can be observed within the groups as well as between the groups in Fig. 1 and Table 2.

In a study by Zhou et al., in a co-study, the effect of conditioned environments of fat-derived stem cells with fractional carbon dioxide laser was investigated for acne atrophy wounds and skin rejuvenation. Nine patients were treated in the skin rejuvenation group and thirteen patients were treated in the acne scar group, and all of peoples underwent three FxCR sessions. This study showed that ADSC-CM with FxCR is a good combination therapy for the treatment of acne atrophic scars and skin rejuvenation [36]. Of course, in some studies, any significant difference was observed in relation to aesthetic results and patients’ satisfaction in SVF injection, but fat accumulation and reabsorption were improved in the SVF-treated groups and beneficial results were observed in this regard [37, 38]. In a similar study, Zhou et al. observed that sebaceous and sweat glands, which are usually absent or sparse at the site of a scar, were found in patients 6 months after SVF nanofat injection. They showed that topical application of SVF-containing medium to skin tissue improves fiber alignment [39].

In addition, a study by Tenna et al. about the comparison of the use of autologous fat grafting with PRP with or without fractional CO2 laser in the treatment of acne scars showed that the improvement of subcutaneous tissue thickness in group A was 0.668 cm and 0.63 cm in group B. All patients in both groups had therapeutic benefits that were confirmed by postoperative FACE-Q module, but no significant difference was observed between the two groups. Finally, this study stated that nanofat subcutaneous injection and PRP seem to be effective in improving atrophic scars, alone or in combination with fractional CO2 laser [40]. In the present study, sonographic findings (epidermal, dermal and complete thicknesses) were improved significantly in the case group than its predecessors and the apparent findings related to visual face including volume, area and depth were significantly different.

Of course, in the control group and in comparison with the baseline, ultrasound findings were significantly different, but the findings of visual face (apparent) despite improving had not statistically significant difference. In addition, in the comparison of the end of the first and third months between the two groups there was no significant difference between the findings. Our findings show that after one month, the apparent variables in the case group had a significant difference from the control group, which shows the significant effect of SVF on the apparent criteria. Also, SVF along with nanofat improves all apparent and sonographic effects of nanofat and makes the effect more and faster, but this effect is more evident in apparent variables compared to sonographic indices.

In general, the findings of our study showed that in terms of scar, sonographic and apparent variables of patients in the SVF group revealed better results than patients in the non-SVF group. In this regard, studies by Lee et al. have shown that in most cases, patients in the SVF group indicated better results than patients in the non-SVF group in scar tissue repair. They reported that subsets of these scales reflected favorable results in terms of height and flexibility. However, there was no significant change in the arteries [41].

The therapeutic performance of SVF injection on skin pores, blemishes, skin clarity, skin melanin content and skin elasticity has also been observed. The results showed that a high percentage of patients reported a good satisfaction after treatment [42]. A systematic review of 665 patients also showed the beneficial effects of SVF-related interventions in the treatment of scarring. Some studies show that SVF injection interventions can be moderately effective in scar healing and are not inferior to PRP or fractional CO2 laser [43]. Past studies have shown that acne can lead to create a variety of prominent and erythematosus scars and influence patients' lives, so it is important to identify new therapeutic strategies [44–46]. Goodarzi et al. stated in their studies that although many therapeutic methods have been used to manage hypertrophic scars, despite the variety of medical interventions, in many cases the therapeutic effectiveness is low and its treatment is still a clinical challenge that requires long-term interventions [47–50]. On the other hand, other findings have shown that paying attention to scar therapeutic options and data collecting about therapeutic methods can help to better choose the therapeutic method [51–53].

The present study showed that the case and control groups in the baseline had not statistically significant difference in terms of apparent and sonographic criteria. Also, at the end of the first month, in the control group, sonographic criteria improved significantly despite the apparent criteria and in the case group, the apparent and sonographic indices had significantly improved. At the end of the third month, only sonographic criteria were examined which in the case and control groups, both dermal and complete thicknesses were significantly better than the baseline, but epidermal thickness was significantly better than the baseline only in the case group. There was no difference between the first and third month in terms of changes in the case and control groups in for sonographic criteria. As a result, SVF enhances and accelerates the positive effects of nanofat treatment, and its positive effect on improving the appearance of scar is more significant than its positive effect on sonographic indices that could be a good complement for nanofat, especially in promoting the effectiveness of scar appearance.

The present study is one of the few clinical trials which evaluates the effect of SVF on acne scar treatment, and compared to them, a longer follow-up period was considered. In this study, the opposite side of the face was also selected as a control, which eliminates the genetic and basic parameters of patients regarding skin repair abilities as confounding variables. However, in this study, no difference was observed between the position of SVF and its effectiveness according to the pilot and low number of patients and re-examination based on the location of scars and the position of SVF product can be effective in optimizing treatment.

## Conclusion

Finally, it can be stated that according to the results of this research, the use of SVF in the treatment of acne scar can significantly improve the total, dermal and epidermal thicknesses, as well as the volume, surface and depth of scar which this difference can be more remarkable in sonographic criteria. However, from the first to the third month, no significant improvement was seen in both groups. In general, SVF injection in acne scar patients can be helpful in controlling and improving scar symptoms. It is suggested that further studies could be performed on a larger population. Also, useful evidences could be obtained by evaluation of psychological and subjective aspects of these patients and cost-effectiveness of this method.

## Data Availability

The data that support the findings of this study are available from the corresponding author [M.A.N] upon reasonable request.
